# Supplementary material for: Extensive 3D mapping of dislocation structures in bulk aluminum
Source: Sci Rep. 2023 Mar 7;13:3834. doi: 10.1038/s41598-023-30767-w (PMC9992398; doi:10.1038/s41598-023-30767-w)
Supplement: Supplementary file 1 — Supplementary Information. [file 41598_2023_30767_MOESM1_ESM.pdf]

Supplementary Information For: Extensive 3D Mapping of Dislocation Structures in Bulk Aluminum

February 8, 2023

1 Dislocation Plane Assignments

Analysis of the dislocation boundary planes and line vectors that was shown in Fig. 4 and Table 1 of the main paper were measured using crystallographic and mathematical tools, as outlined here.

1.1 MSAC Analysis

Using MSAC, we analyzed the 3D `pointCloud` based on the converged value of the probabilistic plane normal fitting. An arbitrary result from MSAC is shown graphically in Figure 1a, with the mean uncertainty measured by the algorithm. To identify the crystallography of the boundary plane, we used transformation matrices to convert our measured orientations in the laboratory coordinate system, $\{x_\ell, y_\ell, z_\ell\}$, to the well-defined crystallographic vectors, $\{x_c, y_c, z_c\}$, that were determined based on the orientations that the single-crystalline samples were cut in, and how they were oriented to place the crystal into the $\{002\}$ Bragg condition.

$$\vec{r}_c = \mathbf{M}^{(\ell\to\mathbf{c})}\tilde{\mathbf{r}}_\ell \tag{1}$$

In this case, our transformation used the crystal cut vectors' uvw vectors that were

$$\mathbf{M}^{(\ell\to\mathbf{c})} = \begin{bmatrix} 1\sqrt{2} & -1\sqrt{2} & 0 \\ 1\sqrt{2} & 1\sqrt{2} & 0 \\ 0 & 0 & 1 \end{bmatrix} \begin{bmatrix} \cos(\theta) & 0 & \sin(\theta) \\ 0 & 1 & 0 \\ -\sin(\theta) & 0 & \cos(\theta) \end{bmatrix}, \tag{2}$$

for which $\theta = 10.38°$ is the X-ray diffraction angle determined from Bragg's law. With the 3D `pointCloud` expressed in the crystal system, the boundary plane was defined as $\vec{n}_c$. Based on $\vec{n}_c$, a third coordinate system, called the boundary-plane system, was then defined such that $z_{\text{bp}} \approx 0$ to reduce the 3D system into a 2D planar one, $\{x_{\text{bp}}, y_{\text{bp}}, z_{\text{bp}}\}$. By selecting 2 arbitrary orthogonal vectors that lie in the boundary plane, $\vec{v}_1$ and $\vec{v}_2$, we defined a second coordinate transformation matrix to convert the crystal system into the boundary-plane system such that

$$\vec{r}_{\text{bp}} = \mathbf{M}^{(c\to\text{bp})}\vec{r}_c, \tag{3}$$

and

$$\mathbf{M}^{(\mathbf{c}\to\text{bp})} = \begin{bmatrix} v_{1x} & v_{1y} & v_{1z} \\ v_{2x} & v_{2y} & v_{2z} \\ \vec{n}_x & \vec{n}_y & \vec{n}_z \end{bmatrix}. \tag{4}$$

As such, Eqation 3 allowed us to transform the 3D `pointCloud` from the 3D crystal system into the boundary-plane coordinate system, where we dropped the $z_{\text{bp}} = 0$ component to dimensionally reduce the system, i.e. $\{x_c, y_c, z_c\} \to \{x_{\text{bp}}, y_{\text{bp}}\}$. The 2D boundary points are shown in Fig. 1b.

To identify the line vector, the 2D boundary points were then rotated to all possible rotational angles (ξ) from 1-180°, and were converted from the 2D boundary system into the candidate 1D dislocation systems via the 2D rotation matrix

$$\vec{r}_d = \mathbf{M}^{(\text{bp}\to\mathbf{d})}\vec{r}_{\text{bp}}, \tag{5}$$

$$\mathbf{M}^{(\text{bp}\to d)} = \begin{bmatrix} \cos(\xi) & -\sin(\xi) \\ \sin(\xi) & \cos(\xi) \end{bmatrix}. \tag{6}$$

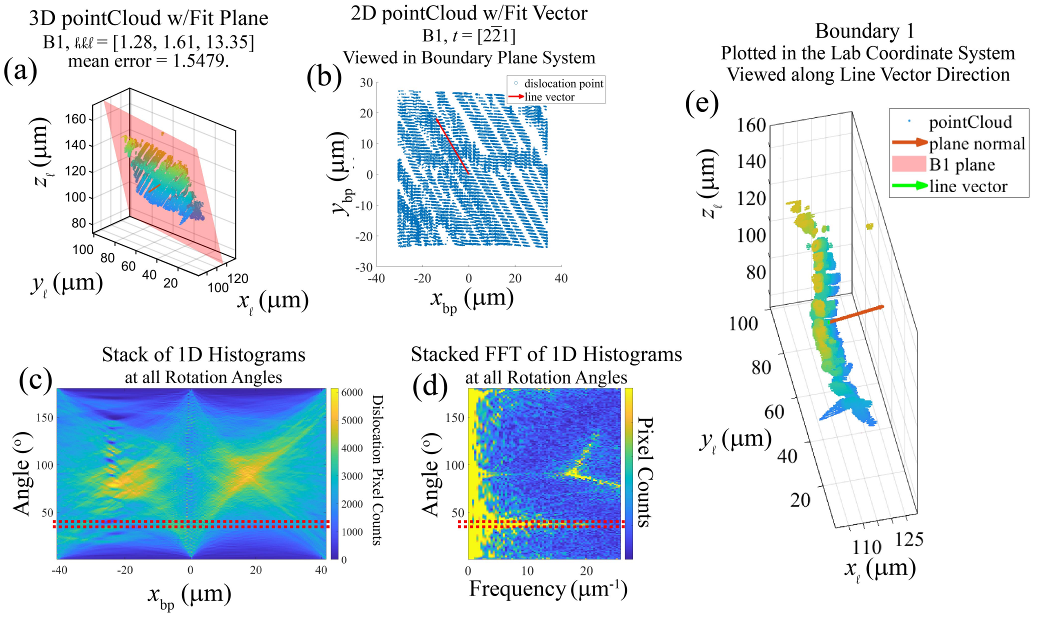

Figure 1: Boundary assignment procedure, showing (a) the plane fitted from the 3D `pointCloud` normal vectors, (b)the 2D representation of the points in the boundary-plane system, (c) an image compiled with each row showing the 1D trace resulting from a histogram of the 2D points, (d) the fast-Fourier transform of the histogram traces, stacked for all angles represented, and (e) the resulting 3D `pointCloud`, as projected along the line vector solved for from the angle identified in (d).**The figure is made using the MATLAB code in the link** `https://github.com/leoradm/Dislocation3DAnalysis.git`

The results of all possible rotations were stacked into the 2D image shown in Figure 1c, and the stack of 1D Fourier transform of those traces are shown in Figure 1d. The features in Fourier space that had the most clearly defined Fourier features corresponded to the ξ_i position that best matched the dislocation line vector, as shown by the red boxes in Fig. 4c-d and the corresponding red vector overlayed on Fig. 1b.

Once the angular offset for the line vector, ξ, was determined, it was converted to the vector defining $\vec{t}_d$ based on

$$\vec{t}_{\rm bp} = [1, \tan(\xi)]. \tag{7}$$

From that result, the dimensions were restored into 3D by adding the implicit $z_{\rm bp} = 0$ back into the arrays, and inverting all transformation matrices as

$$\vec{r}_c = \mathbf{M}^{(\ell \to \mathbf{c})^{-1}} \mathbf{M}^{(\mathbf{c} \to {\rm bp})^{-1}} \vec{r}_{\rm bp}, \tag{8}$$

to plot the full 3D line vector in the lab system, overlaid on the 3D `pointCloud` plot and was verified as the line vector by viewing the system along the $\vec{t}_\ell$ vector, as shown in Figure 1e. We note that the correct line-vector assignment was evident because all dislocations aligned into well-defined points along the viewing axis. From this point, we converted the solved $\vec{t}_{uvw}$ vectors from the crystal system into the corresponding $\vec{t}_{hkl}$ values, then manually rounded the values to their nearest integers and graphically verified that the assignments were accurate.

2 Context on Spatial Resolution and Crystallographic Defects with other DFXM Modalities

Fig 2(a) shows a resolution target having 150 nm line spacing with 88 Be CRLs used for this experiment at 17 keV in bright field mode (i.e. $2\theta = 0°$). Fig 2(b) shows the focused line beam on the farfield camera (bright field mode) and Fig 2(c) shows the intensity profile of the line focus, having a 550 nm FWHM.

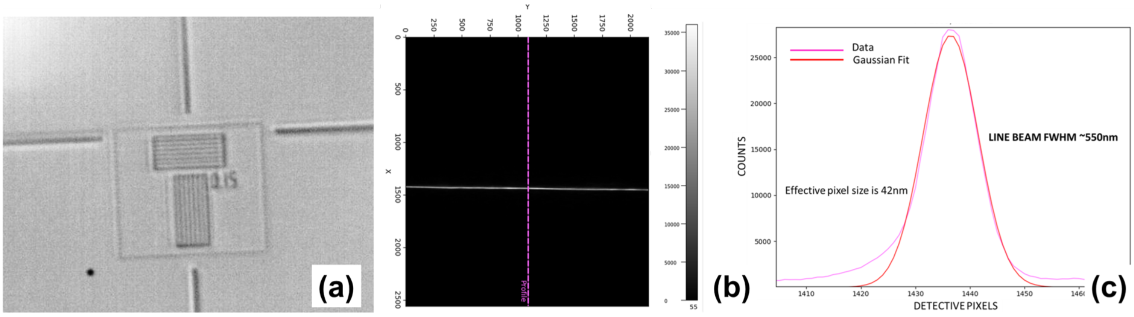

Figure 2: a 150 nm Resolution Target in the bright field mode (a) 1D focused line beam in the bright field mode (c) intensity profile of the line focused beam. **The figure is made using the MATLAB code in the link** https://github.com/leoradm/Dislocation3DAnalysis.git and https://gitlab.esrf.fr/julia.garriga/darfix

Fig. 3 shows the weak and strong beam cases using box-shaped beam having a size of 300 μm $\times$ 300 μm . Fig. 3(a) shows a network of dislocations visible at WB contrast. Although these type of scans show a projection of the 3D structure in the scanned volume, there is no height information grain in this as to where these dislocations are positioned in 3D.

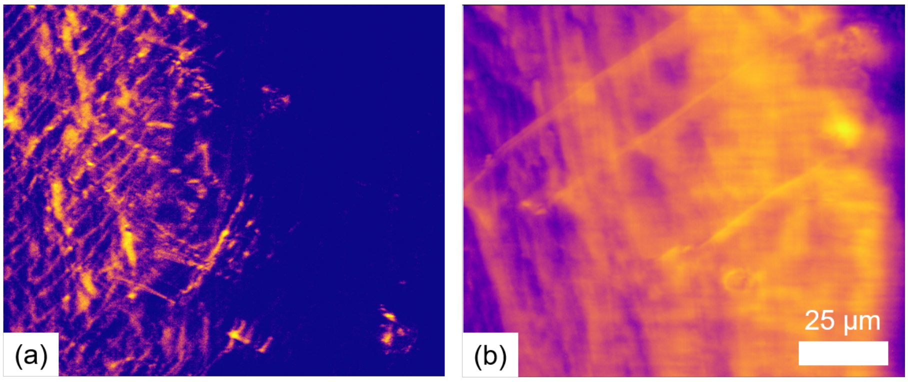

Figure 3: Box-beam projection images of (a) weak-beam (b) strong-beam conditions. **The figure is made using the MATLAB code in the link** https://github.com/leoradm/Dislocation3DAnalysis.git and https://gitlab.esrf.fr/julia.garriga/darfix

Fig 4(a-d) shows the ϕ COM maps selected slices of a layered mosaicity scan that spans 40 μm in z direction. Fig 4(e) shows COM map generated from the projection scan (i.e box beam case) of the same volume using the 2$\times$ objective.

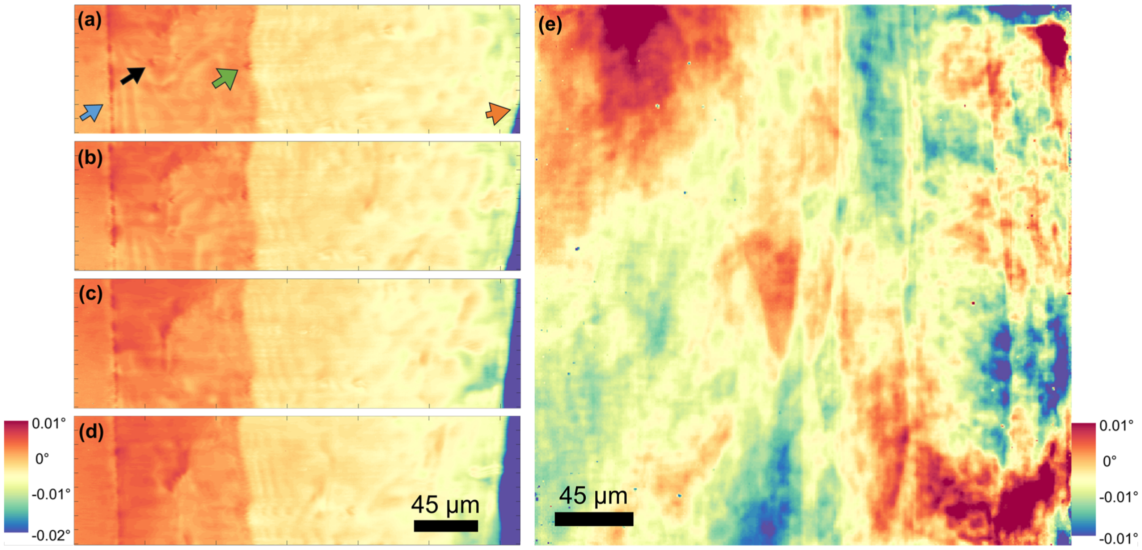

Figure 4: 2D sections of center of mass maps of sample orientation. The spacing between the layers are 5μm. The black arrow in (a) highlights the dislocation structures in the orange subgrain. The other arrows mark the dislocation boundaries. **The figure is made using the MATLAB code in the link** https://github.com/leoradm/Dislocation3DAnalysis.git and https://gitlab.esrf.fr/julia.garriga/darfix

3 Supplementary Videos

We have included two videos to show the 3D heterogeneity of the orientation distribution. The first video shows the generated mosaicity maps (i.e. orientation maps collected using the two sample tilts, ϕ and χ), of 20 layers with 1 μm distance between each layer. The video titled "Layered Mosaicity" shows different subgrains separated by dislocation boundaries with low misorientation. The color coding of this video is represented in the second video (titled "Orientation distribution colormap of the layered mosaicity map" which shows the orientation distribution (i.e. local reciprocal space map) of the same 20 layers. Both of these videos are made using the MATLAB code in the link https://github.com/leoradm/Dislocation3DAnalysis.git and https://gitlab.esrf.fr/julia.garriga/darfix
